# Point-of-Care Prostate Specific Antigen Testing: Examining Translational Progress toward Clinical Implementation

**DOI:** 10.1021/acssensors.3c01402

**Published:** 2023-10-13

**Authors:** Saweta Garg, Ashwin Sachdeva, Marloes Peeters, Jake McClements

**Affiliations:** † Merz Court, School of Engineering, Newcastle University, Claremont Road, NE1 7RU Newcastle upon Tyne, U.K.; ‡ Division of Cancer Sciences, University of Manchester, Wilmslow Road, Manchester M20 4BX, U.K.; § Department of Urology, The Christie NHS Foundation Trust, Manchester M20 4BX, U.K.; # Department of Chemical Engineering and Analytical Science, School of Engineering, University of Manchester, Manchester M20 4BX, U.K.

**Keywords:** prostate cancer, prostate specific antigen, point-of-care testing, translational progression, biosensors, electrochemical detection, optical detection, healthcare devices

## Abstract

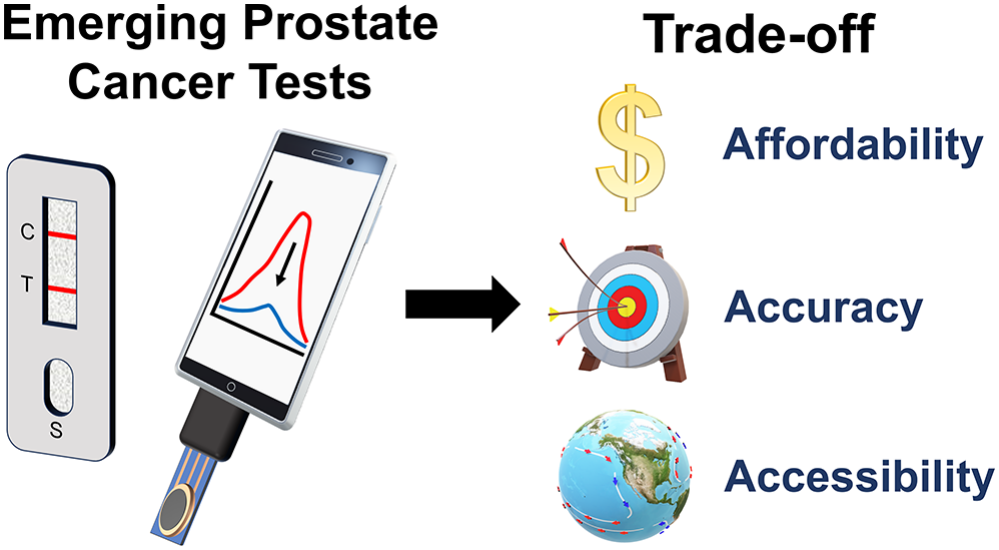

Prostate cancer (PCa) is the second most common male cancer and is attributable to over 375,000 deaths annually. Prostate specific antigen (PSA) is a key biomarker for PCa and therefore measuring patient PSA levels is an important aspect of the diagnostic pathway. Automated immunoassays are currently utilized for PSA analysis, but they require a laboratory setting with specialized equipment and trained personnel. This results in high diagnostic costs, extended therapeutic turnaround times, and restrictions on testing capabilities in resource-limited settings. Consequently, there is a strong drive to develop point-of-care (PoC) PSA tests that can offer accurate, low-cost, and rapid results at the time and place of the patient. However, many emerging PoC tests experience a trade-off between accuracy, affordability, and accessibility which distinctly limits their translational potential. This review comprehensively assesses the translational advantages and limitations of emerging laboratory-level and commercial PoC tests for PSA determination. Electrochemical and optical PSA sensors from 2013 to 2023 are systematically examined. Furthermore, we suggest how the translational potential of emerging tests can be optimized to achieve clinical implementation and thus improve PCa diagnosis globally.

Prostate cancer (PCa) is the second most common male cancer globally.^1^ In 2020, there were approximately 1.4 million new cases worldwide and over 375,000 deaths attributable to PCa. The average age of diagnosis is 66 years, and cases are seldom identified in individuals below 40 years.^2^ Since the late 1990s, the incidence of PCa has risen by 3–7% annually, with gradual improvements in survival rates.^3,4^ Although this trend is attributable to multiple factors, it is proposed that the widespread introduction of prostate specific antigen (PSA)-based testing in the early 1990s had a significant influence.^5,6^ This is because PSA tests allow for earlier PCa diagnosis and can predict cancer recurrence, which significantly improves patient outcomes. For example, before widespread PSA testing was introduced, 30–35% of men had bone metastasis when PCa was diagnosed, and mortality rates ranged from 20 to 60%, depending on the country.^7^ Today, approximately 5–7% of men present with metastatic PCa, and 5-year survival rates are over 97%.^8,9^ However, despite the clear benefits of PSA testing, there are several drawbacks related to current clinical practice and technologies.

PSA is a 30–34 kDa kallikrein-related peptidase secreted by benign and cancerous prostate epithelial cells. PSA degrades gel-forming proteins in the ejaculate, resulting in the liquefaction of semen and the release of motile sperm. While normally confined within the prostate, small quantities of PSA can be detected in semen and blood. In serum, a minor fraction of PSA is present in its free form (fPSA), while the predominant molecular form is an 80–90 kDa complex of PSA with α1-antichymotrypsin.^10^ PCa alters the microarchitecture within the prostate, which leads to higher quantities of PSA leaking out into the blood. Men with a serum PSA level between 4 and 10 ng/mL have a 25% chance of having PCa, which increases to 50% when PSA levels are above 10 ng/mL.^11^ However, while serum PSA is widely used for PCa diagnosis and monitoring recurrence after treatment, it can be difficult to reliably interpret since PSA levels tend to rise with advancing age as well as in other conditions such as urinary tract infections (UTIs), benign prostatic hyperplasia, and prostatitis. This leads to a high false-positive rate, with 75% of men with a raised PSA level subsequently found to have a negative prostate biopsy.^12,13^ Furthermore, PCa can also be detected in men without raised PSA levels.^14^ This leads to false-negative findings since PCa is diagnosed in up to 15% of men with PSA levels below 4 ng/mL.^15^ Conversely, PSA testing may also result in overdiagnosis of clinically insignificant PCa, which has limited potential to cause significant harm during the man’s lifetime.^5^ Studies have estimated that 40–60% of PCa detected using PSA testing may be clinically insignificant.^16,17^ Such overdiagnosis led to approximately 1.4 million men within the US receiving unnecessary treatment for PCa between 1986 and 2005.^18^

Given that PSA levels increase with advancing age, the use of age-adjusted PSA thresholds are advised to minimize overinvestigation.^19^ Men with a raised PSA are referred for specialist urological review and increasingly undergo an MRI scan of the prostate to identify radiologically concerning prostate lesions, which are categorized using the prostate imaging reporting and data system (PI-RADS) criteria.^20^ To improve the detection of clinically significant disease while minimizing the detection of clinically insignificant PCa, the American Urological Association (AUA) recommends that multiparametric prostate MRI (mpMRI) be used as part of the diagnostic workflow. This involves undertaking biopsies of MRI-positive (PI-RADS ≥ 3) prostate lesions in a systematic and targeted manner.^21^ However, the cost of MRI scans is high (∼$400–$10,500), and reporting turnaround time may vary from a few days to a few weeks, with limited availability in developing countries.^22,23^

Prostate biopsies are performed by visualizing the prostate using transrectal ultrasound and extracting multiple tissue core samples of the prostate under local anesthesia.^24^ These can be taken by passing a core biopsy needle through either the rectum or the perineum to target various regions of the prostate, focusing primarily on the peripheral zone where PCa most frequently arises. Prostate biopsies are associated with numerous complications, including pain, bleeding (e.g., hematospermia, hemeaturia, hematochezia), infection (e.g., cystitis, prostatitis, epididymorchitis, urosepsis), transient erectile dysfunction, and difficulty passing urine (including urinary retention).^25−27^ Urinary retention, UTIs, and sepsis account for emergency room visits and hospitalization in 2–4% of cases, with severe urosepsis resulting in a small but significant number of admissions to intensive care units (∼0.3%) and deaths (∼0.1%).^27,28^ False-negative results are also still common (15–20% occurrence).^29,30^ Moreover, given the high number of prostate biopsies performed, false-positive results based upon raised PSA levels place significant financial burdens on healthcare systems. For example, in the US, 1.5 million biopsies are performed yearly, equating to annual costs of $2.5 billion.^31^

Despite sensitivity and specificity issues, PSA tests have significantly reduced overall PCa mortality.^6,32^ Furthermore, PSA testing plays a crucial role in the early detection of PCa recurrence, which occurs in up to 25% of patients following radical treatment.^33^ Currently, PSA tests are performed at a medical facility (mostly primary care) and sent to laboratories for analysis, which is typically carried out using automated immunoassays. The advantages of these automated analyzers include low limits of detection (LoD), reliability, and high throughput of samples. However, they require specialist equipment, trained personnel, and rapid sample transportation (blood samples must reach the laboratory within 16 h).^34,35^ Consequently, this analysis is not readily available in community or resource-limited settings. Furthermore, patients often wait ∼1–2 weeks for PSA test results, and the assays are relatively expensive (∼$19).^36^ Consequently, there is a major clinical need for point-of-care (PoC) PSA tests that can provide rapid, low-cost, and reliable results at the time and place of the patient and therefore help expedite the pathway for diagnosis or disease monitoring. Recent advances in nanotechnology have enabled the development of miniaturized microfluidic techniques for analyte detection, which have the potential to revolutionize medical diagnostics in this setting.

In this review, we will critically summarize the literature from 2013 to 2023 on PoC sensors for PSA detection and investigate their advantages, limitations, and suitability for widespread use. Unlike other reviews in this area, we will focus on the translational progression of the technology to identify the challenges that laboratory-level PoC tests must overcome to enable their widespread adoption across healthcare services. This will provide a comprehensive assessment of the clinical potential of emerging PoC technology, which will help to guide translational progression and thus ultimately improve future PCa diagnosis.

## Demand for PSA Testing

There is a high global demand for PSA testing for population screening, diagnosis, and post-treatment monitoring. Each year within the US, tests are performed on 13% of men aged 40–54 years and 39% of men aged 55–69 years.^37^ Screening for PCa is among the most controversial topics in the urological literature. Population-based PCa screening with PSA tests was widely adopted within the 1990s, which decreased mortality and also led to significant overdiagnosis and unnecessary treatment. Current guidance for primary care physicians in the UK, US, and Australia recommends discussing and coming to a shared decision regarding PSA testing with men who raise the issue or have certain risk factors.^38,39^ However, this passive advice has led to widely variable testing rates across different countries and even between different medical sites. Furthermore, although these measures have reduced overdiagnosis, they have also been problematic in some countries. For example, in the US, the number of men with metastatic PCa upon diagnosis has increased by approximately 50% since PSA screening was abolished.^40^ Thus, a clear need remains for strategic screening using a combination of tools, including PSA testing, particularly as the global population is rapidly aging and PCa-specific mortality strongly correlates with increasing age.^1^ This has resulted in guidelines for screening and early detection of PCa that recommend using a combination of PSA testing, mpMRI, and a risk calculator for biopsy indication in asymptomatic men with a PSA level between 3–10 ng/mL and a normal digital rectal exam.^41^

While screening for PCa might be controversial, PSA testing is widely recommended for the early detection of PCa recurrence. In the US, the National Comprehensive Cancer Network (NCCN) recommends PSA testing every 6 to 12 months for 5 years after PCa treatment, followed by yearly monitoring.^33^ After radical prostatectomy for PCa, 3–26% of patients experience biochemical recurrence (BCR), which is defined as PSA values persistently > 0.2 ng/mL.^42−,201^ Overall, this confirms the high demand for PSA testing for screening, early diagnosis, and recurrence of PCa, which will continue to increase due to an aging population. Moreover, there is an unmet need for reliable PoC PSA tests in resource-limited settings, as access to equipment utilized in the PCa diagnostic pathway (e.g., automated immunoassays, mpMRI) is severely restricted in these locations.

## Current Testing Methods

Within healthcare systems, there is a wide array of automated immunoassays utilized for PSA measurements, including chemiluminescent immunoassays (CLEIA), electrochemiluminescence immunoassays (ECLIA), and chemiluminescent magnetic microparticle immunoassays (CMIA). Currently, most PSA tests are conducted in centralized laboratories using large, automated high throughput immunoanalyzers.^43^ The major companies that manufacture these analyzers are Abbott Diagnostics (Alinity I), Beckman Coulter Access (Access Dxl), Roche Diagnostics (Cobas e801), and Siemens Healthcare Diagnostics (Atellica IM). These immunoassays offer numerous benefits, such as low LoDs ranging from 0.003–0.02 ng/mL and high sample throughput (∼100 tests/hour).^44^ However, they require dedicated laboratories, which prevents their widespread use in resource-limited settings, and have long turnaround times (duration between testing and taking therapeutic action).^34,35^

Another significant issue with current PSA testing methods is that different automated immunoassays can have large discrepancies in their results due to variations in recognition elements (e.g., antibody types), apparatus, and testing specifications. To overcome the interassay variability across manufacturers, the World Health Organization (WHO) enforced two standards for PSA testing in 1994. The WHO 96/670 standard is for the calibration of total PSA (tPSA) tests (contains an equimolar ratio of 90:10 of tPSA to fPSA), and the WHO 96/668 standard is for fPSA tests (100% fPSA).^45^ Despite improvements driven by the introduction of WHO standards, reports suggest that results from tPSA tests using different automatic analyzers still have significant discrepancies.^46,47^ Furthermore, there are even wider disagreements among different fPSA tests, thus questioning the viability of their clinical use. These issues can significantly impact the clinical interpretation of PSA tests, which can lead to misinformed treatment decisions.^44^ Manual ELISA immunoassays are still widely employed for PSA testing in developing countries, such as the AccuBind Total PSA Test (Monobind). However, Murthy et al. showed extremely poor agreement between five different ELISA-based manual PSA test kits, which further affirms the need for accurate PoC tests in developing countries.^48^

Several next-generation assays have also been developed for PCa diagnosis. For example, the Stockholm3 test measures five blood biomarkers (including PSA), over 100 genetic markers, and clinical features (e.g., age and family history). This information is then combined to generate a personalized risk score for PCa, which can be used for treatment decisions.^39^ Other next-generation assays include the Prostate Health Index test, which measures multiple biomarkers (fPSA, tPSA, [-2]pro-PSA), and the 4Kscore test, which combines biomarkers (fPSA, intact PSA, tPSA, kallikrein-like peptidase 2[hk2]) with age, digital rectal examinations, and prior biopsy results. However, these methods are high-cost (Stockholm3 is ∼$450/test), making them unsuitable for widespread use, particularly in resource-limited environments.^22,49^

## Emerging Point-of-Care Tests

There are numerous drawbacks associated with the PSA testing methods currently employed across healthcare systems. Consequently, there is widespread focus on developing alternative PoC tests for PSA detection. However, while these tests may perform favorably at the laboratory level, many benchmarks must be met to enable translation to healthcare settings. For example, the WHO has specified that emerging PoC diagnostics should meet the ASSURED criteria (Affordable, Sensitive, Specific, User-friendly, Rapid, Equipment-free, and Deliverable to end-users).^50^ In 2019, Land et al. updated these criteria to include Real-time connectivity and Ease of specimen collection, resulting in REASSURED.^51^ The following section presents and critically evaluates emerging laboratory-level PoC tests for PSA detection in relation to their translational potential. For ease of comparison, the tests are separated into electrochemical and optical detection. Furthermore, several recently developed PoC commercial PSA tests are also presented and assessed. Due to the popularity and rapid advancements of this field, only literature from 2013 to 2023 will be assessed.

### Electrochemical Detection Methods

Electrochemical detection is based on measuring changes in an electrical signal, which occur when target analytes interact with a sensing surface.^52^ This typically involves a three-electrode system comprising working, counter, and reference electrodes. The working electrode acts as the transducer element, the counter electrode closes the current circuit within the electrochemical cell, and the reference electrode maintains an established potential.^53^ For the determination of PSA, either label-free or label-based electrochemical detection is employed. Label-free detection measures changes in electrochemical current or impedance and correlates this to the PSA concentration. This can be performed via modification of the working electrode surface with an electrically conductive material (e.g., Au, carbon) or via the inclusion of an external redox probe (e.g., [Fe(CN)_6_]^3–/4–^).^54^ Label-based detection is an indirect method that detects a labeling compound (e.g., nanoparticles (NPs), carbon nanomaterials) tagged to the PSA molecules.^55^ A multitude of electrochemical readout methods can be utilized for PSA detection, with the most common being electrochemical impedance spectroscopy (EIS), differential pulse voltammetry (DPV), cyclic voltammetry (CV), square wave voltammetry (SWV), linear sweep voltammetry (LSV), and chronoamperometry (CA). Electrochemical methods are commonly employed for PSA detection and have significant potential for commercial PoC testing due to their excellent sensitivity/specificity, low-cost, ease of operation, and simple integration into portable devices.

Numerous recent electrochemical PSA tests from the literature are listed in Table 1. To assess translational potential for PoC testing, several critical factors were identified based on the REASSURED criteria.^51^ These include sensor materials, the readout method, assay time, sample volume, linear range, LoD, and sample type. The most commonly utilized materials for the working electrode are Au and glassy carbon due to their smooth surfaces and electrical conductivity, which improve sensor performance.^56,57^ For example, Rafique et al. immobilized PSA antibodies to polymer brush-modified Au electrodes and utilized EIS for detection. The sensor exhibited an excellent linear range (0.005–1000 ng/mL) and LoD (0.002 ng/mL), but PoC potential is unclear as no assay time or sample volume was provided, and measurements were only performed in buffered solutions.^58^ Mwanza et al. also utilized EIS for PSA measurements with antibodies immobilized to isophthalic acid-grafted Au electrodes.^60^ A very wide linear range (1 × 10^–5^–100 ng/mL) and low LoD (3.3 × 10^–6^ ng/mL) were achieved, but measurements were not performed using clinical samples. Furthermore, the assay time for PoC PSA tests should be ∼20 min to allow for a test and subsequent discussion of results to be performed within the same clinical session.^59^ However, the test developed by Mwanza et al. had an assay time of 60 min, which may limit translational potential.

**Table 1:** Summary of Electrochemical Sensors for PSA Detection^a^

Sensor Materials/Capture Probe^b^	Readout Method^c^	Assay Time (min)	Sample Volume (μL)	Linear Range (ng/mL)	LoD^d^(ng/mL)	Samples	Ref
IPA/Au	EIS	60	-	1 × 10^–5^to 100	3.3 × 10^–6^	Standard solution	(60)
Ab							
GRP-polymer/Au	EIS	5	10	0.0001–100	4 × 10^–5^	Standard solution	(61)
Ab						Spiked saliva	
l-Cys/Au	CV	-	3000	0.0002–0.0009	0.0001	Standard solution	(57)
Ab							
PNIPAAm/Au	Amperometry	35	50	0.01–10	0.0009	Standard solution	(62)
Ab						Patient serum	
-MWCNTs-IL-Chit/GCE	DPV	55	-	0.05–80	0.001	Standard solution	(63)
Ab						Spiked serum	
Polymer/Au	EIS	-	-	0.005–1000	0.002	Standard solution	(58)
Ab							
Au/Pt	Ampero-metry	30	10	0.01–1.5	0.002	Standard solution	(64)
Ab						Patient serum	
IDC sensor chip	IDC	1	-	0.1–100 μL/mL	0.1 μL/mL (semen)	Human semen	(65)
Ab							
Au	EIS	60	60–360	1–10	0.118	Standard solution	(56)
Ab						Spiked serum	
AuNF/SPCE	CV	15	100	0.1–100	0.28	Standard solution	(66)
Ab					1.84 (serum)	Patient serum	
GO/Au	DPV	60	500	1–100	1	Standard solution	(67)
Ab						Patient serum	
AuNP-cells/SPCE	CA	5	50	0.5–10	1.72	Standard solution	(68)
Ab						Patient serum	
MWCNTs-PAMAM/GCE	LSV	40	15	0.001–30	0.0007	Standard solution	(69)
Peptide						Spiked patient serum	
AuNP/PWE	DPV	40	40	0.002–40	0.001	Standard solution	(70)
Peptide							
SF-TiO_2_/GCE	EIS	40	15	2.5 × 10^–6^ to 25	8 × 10^–7^	Standard solution	(71)
Aptamer/MCH						Spiked serum	
MoS_2_/GCE	SWV	120	12	1 × 10^–6^ – 500	2.5 × 10^–6^	Standard solution	(72)
Aptamer						Patient serum	
Au	LSV	60	-	0.001–160	0.0001	Standard solution	(73)
Aptamer						Patient serum	
Au	EIS	-	100	0.1–100	0.001	Standard solution	(74)
Aptamer–MIP							
HL-Au	DPV	60	-	0.125–128	0.04	Standard solution	(75)
Aptamer						Patient serum	
Au/Ti IDE	EIS	70	50	0.5–5000	0.51 (serum)	Spiked serum	(76)
Aptamer							
Au-GO/GCE	EIS	35	-	0.5–7	0.85	Standard solution	(77)
Aptamer							
Au/Si	SWV	21	-	1–100	1.1	Standard solution	(78)
Aptamer							

a Unless stated otherwise, the presented LoD values are for measurements performed in standard solution.

b Antibody (Ab); Chitosan nanocomposite (Chit); Glassy carbon electrode (GCE); Graphene oxide (GO); Graphene (GRP); Hairbrush-like gold nanostructure (HL-Au); Interdigitated capacitor (IDC); Interdigitated electrode (IDE); Ionic liquid (IL); Isophthalic acid (IPA); l-Cysteine (l-Cys); 6-mercapto-1-hexanol (MCH); Molecularly imprinted polymer (MIP); Molybdenum disulfide (MoS_2_); Multiwalled carbon nanotubes (MWCNTs); Nanoflowers (NF); Nanoparticle (NP); Poly(amidoamine) (PAMAM); Poly(*N*-isopropylacrylamide) (PNIPAAm); Paper working electrode (PWE); Silk fibroin nanofiber (SF); Screen-printed carbon electrode (SPCE).

c Chronoamperometry (CA); Cyclic voltammetry (CV); Differential pulse voltammetry (DPV); Electrochemical impedance spectroscopy (EIS); Linear sweep voltammetry (LSV); Square wave voltammetry (SWV).

d Limit of detection (LoD).

Many reports also utilize glassy carbon electrodes (GCEs) within electrochemical PSA sensors. For example, Kavosi et al. developed GCEs modified with multiwalled carbon nanotubes (MWCNTs), ionic liquid, and poly(amidoamine) dendrimers. Antibodies acted as the recognition element and DPV was employed as the readout method, which facilitated PSA measurements across a clinically relevant range (0.05–80 ng/mL) with a LoD of 0.001 ng/mL.^63^ Nevertheless, only four measurements were performed on spiked serum samples, and the assay time was long (50 min) for PoC applications. While biosensors containing Au electrodes and GCEs generally demonstrate excellent PSA detection, both materials are relatively expensive, which significantly limits their potential for widespread use within low-cost and disposable sensors.^79^

**Figure 1:**
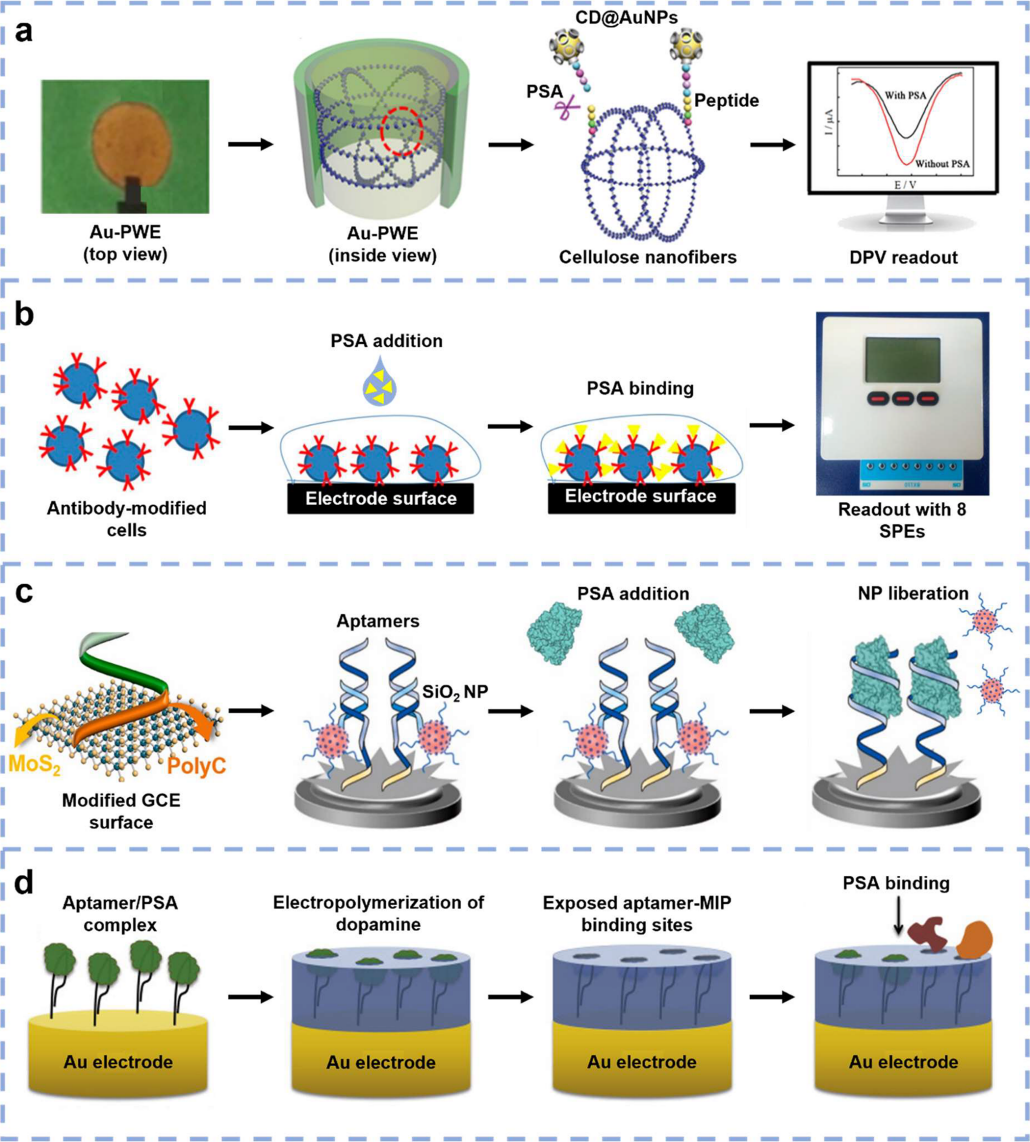
Emerging sensors for electrochemical detection of PSA. (a) Peptides with functionalized AuNPs are immobilized on Au/paper-based working electrodes (PWEs). PSA introduction cleaves the peptides, which removes AuNPs and, thus, reduces the DPV signal. Adapted with permission from (70). Copyright 2018, American Chemistry Society. (b) PSA antibodies are electroinserted into Vero cells, which are then immobilized to AuNP-modified screen-printed carbon electrodes. Eight electrodes were inserted into a portable device for simultaneous CA measurements. Adapted from ref (68). Creative Commons CC BY license, MDPI. (c) Aptamers are immobilized to molybdenum disulfide-modified GCEs, and Si nanoprobes with electroactive tags are utilized for signal amplification. Introducing PSA liberates the Si nanoprobes and therefore reduces the SWV signal. Adapted with permission from ref (72). Copyright 2022 Elsevier. (d) Aptamers/PSA are immobilized to Au electrodes before electropolymerization of a dopamine layer. The PSA is then removed, which leaves exposed binding sites to allow direct detection using EIS. Adapted from ref (74). Creative Commons CC BY license, Elsevier.

To improve PoC testing potential, several studies have modified low-cost working electrodes with nanomaterials (e.g., NPs) to achieve high performance without a considerable cost. For example, Zheng et al. modified paper-based working electrodes with AuNPs, which is a low-cost option compared to using a fully Au electrode. A peptide was utilized as the recognition element, where one end was immobilized to the working electrode, and cyclodextrin-functionalized AuNPs were immobilized to the other. Introducing PSA caused the peptide to break, which stripped the functionalized AuNPs from the working electrode. This led to measurable changes to the DPV signal, which increased with PSA concentration (Figure 1a). The study presented a promising PoC test that is low-cost and portable with a clinically relevant LoD (0.001 ng/mL) and linear range (0.002–40 ng/mL). However, patient samples must be measured to fully assess the sensor’s clinical potential and strategies to reduce assay time (40 min) should be explored.^70^

Mavrikou et al. also explored low-cost electrodes by developing AuNP-modified screen-printed carbon electrodes (SPCEs). In recent years, SPCEs have become popular sensor components due to their favorable performance, suitability for mass production, and low-cost (<$0.1).^80,81^ Within the study, a cell-based biosensor was developed by electroinserting antibodies into Vero cells and immobilizing them onto AuNP-modified SPCEs (Figure 1b). Eight CA measurements were performed in parallel using a portable potentiostat, where cell membrane potential decreased due to PSA binding. The test was rapid (5 min) and only required 50 μL of sample fluid. Furthermore, measurements of 39 patient samples were successfully benchmarked against an immunoradiometric assay and the test cost was estimated at <$12. However, the sensitivity was limited as the LoD (1.72 ng/mL) and linear range (0.5–10 ng/mL) do not fully encompass clinically relevant levels. Membrane-engineered cells also have low stability, which severely restricts shelf life and storage conditions. These drawbacks suggest the technology is unlikely to be used for PoC measurements.^68^ Dou et al. also developed SPCE-based tests for PSA detection. The SPCEs were modified with Au nanoflowers and incorporated into lateral flow assays (LFAs), where PSA binding was measured using CV via a smartphone-based device. The study achieved an improved LoD (0.28 ng/mL) compared to Mavrikou et al. without a significant compromise on measurement time (15 min) or sample volume (100 μL). Moreover, LFAs are already widely used within PoC diagnostics, and the smartphone-based readout device is easy to operate and portable. Consequently, the study presents a test with considerable translational potential.^66^

The recognition element used within PSA tests can also impact the PoC potential. Most tests use antibodies as recognition elements as they exhibit high sensitivity/specificity toward PSA. For example, Ortega et al. immobilized antibodies to Au/Pt-coated electrodes to detect PSA using a smartphone-based sensor, which exhibited a favorable LoD (0.002 ng/mL), reasonable measurement times (30 min), and low sample volume requirements (10 μL).^64^ However, antibodies are laborious and expensive to produce, frequently exhibit high batch-to-batch variation, and have poor environmental stability.^80,82^ Due to these issues, several next-generation recognition elements have become more common within emerging PSA sensors. Aptamers (short single-stranded DNA or RNA molecules) are the most widespread alternative recognition element due to their high binding affinity, excellent stability, limited batch-to-batch variation, and relatively low-cost.^54,83^ Sattarahmady et al. immobilized aptamers to Au electrodes and PSA levels were measured using DPV. A high sensitivity (LoD = 0.04 ng/mL) and wide linear range (0.125–128 ng/mL) were achieved, and measurements of 10 patient serum samples were successfully benchmarked using an immunoradiometric assay. However, translational potential is limited due to long measurement times (60 min) and high-cost electrodes.^75^ Yan et al. also developed an aptasensor for PSA detection, in which aptamers were anchored to molybdenum disulfide-modified GCEs. Si nanoprobes were tagged to the aptamer for signal amplification, and SWV was utilized as the readout method (Figure 1c). The sensor was validated using 12 patient serum samples and exhibited an exceptional LoD (2.5 × 10^–6^ ng/mL) and linear range (1 × 10^–6^–500 ng/mL). Despite the excellent sensor performance, translational potential is also limited due to very long measurement times (120 min) and costly electrode materials.^72^

Benvidi et al. developed an aptasensor for PSA detection using a GCE modified with AuNPs and silk nanofiber/TiO_2_ nanocomposites, which exhibited the lowest LoD (8 × 10^–7^ ng/mL) of any test within Table 1. Furthermore, the aptasensor had low sample volume requirements (15 μL) and measurements of spiked patient serum (n = 4) were benchmarked with good agreement against a CMIA. Although the test exhibited excellent performance, the aptasensor also had relatively long measurement times (40 min) and high-cost electrode materials.^71^ Aptamers have also been immobilized to Au/Ti interdigitated electrodes for PSA detection using Faradaic mode EIS. The sensor exhibited a clinically relevant linear range (0.5–5000 ng/mL) and LoD (0.51 ng/mL in serum) in addition to having low sample volume requirements (15 μL). Moreover, the authors state that the sensor is low-cost compared with other emerging PSA sensors containing carbon nanomaterials. However, as with all emerging aptasensors for PSA, the long assay time (70 min) significantly hinders the potential for PoC applications.^76^

Molecularly imprinted polymers (MIPs) are also promising alternative recognition elements for PSA detection, as they are low-cost, highly stable, suitable for mass production and exhibit high binding affinity. MIPs are polymer matrices with imprinted cavities that match the target molecule’s size and shape.^52^ Jolly et al. added aptamers to the imprinted cavities of MIPs to develop hybrid apta-MIP recognition elements (Figure 1d). EIS was utilized to directly detect PSA in a clinically relevant range (0.1–100 ng/mL) with a favorable LoD (0.001 ng/mL). The sensor showed high promise, but measurements were only performed in buffered solutions and assay time was not mentioned.^74^ Peptides have also been utilized as recognition elements for PSA detection due to their high environmental stability, simple synthesis process, low-cost, and excellent sensitivity.^84^ He et al. developed a PSA biosensor by immobilizing peptides to GCEs modified with MWCNTs and poly(amidoamine) dendrimers. Dithiobis(succinimidylpropionate) Au@SiO_2_ nanohybrids, which acted as tracing tags, were then covalently bound to the free end of the peptide. Introducing PSA caused the peptide to break, which reduced the intensity of the LSV signal. The sensor exhibited an excellent linear range (0.001–30 ng/mL) and LoD (0.0007 ng/mL), in addition to a low sample volume (15 μL). However, PoC potential may be limited due to high-cost sensor components (e.g., GCEs and MWCNTs) and relatively long measurement times (40 min).^69^

To comprehensively assess the translational potential of PSA tests, measurements must be performed using patient samples and benchmarked against standard immunoassays. Most tests within Table 1 perform clinical measurements but typically only using a small number of patient samples (median of 5.5). However, Dou et al. and Ortega et al. performed measurements using a higher number of patient serum samples (n = 46 and n = 60, respectively). Ortega et al. validated their results against gold standard ELISA and radioimmune assays with excellent correlation (95% confidence interval).^64^ The patient samples used by Dou et al. were previously measured with ELISA and their results demonstrated significant differences between PCa negative and positive samples using one-way analysis of variance (***p < 0.001). These clinical measurements, coupled with low-cost electrodes, rapid assay times, and favorable detection, further affirm the PoC potential of this emerging PSA sensor.^66^

Some studies have also explored alternative sample fluids for PSA measurements. For example, Mishra et al. developed a low-cost biosensor (<$5) for PSA detection in semen using an antibody-functionalized interdigitated capacitor. The test had very short measurement times (1 min) and excellent sensitivity in a clinically relevant range for semen (0.1–100 μL/mL).^65^ Another study immobilized antibodies to graphene/polymer-modified Au electrodes for PSA detection in saliva. Within buffered solutions, the sensor exhibited a very wide linear range (0.0001–100 ng/mL) and excellent LoD (4 × 10^–5^ ng/mL). Furthermore, measurements in spiked saliva (n = 6) showed a high correlation with ELISA (94% linearity), and the sensor exhibited rapid assay times (5 min) and low sample volume requirements (10 μL).^61^ Saliva may be a promising sample fluid for PSA testing due to its noninvasive collection, quick processing, and cost-effectiveness compared to traditional blood draws. Moreover, emerging studies have established a direct correlation between PSA levels in saliva and blood, especially in patients with recurrent or metastatic PCa.^61^ This correlation underscores the potential for saliva-based PSA tests as an alternative diagnostic tool. However, the limited utilization of saliva-based PSA tests within healthcare systems can be attributed to several factors. For example, although research correlating PSA levels in saliva and blood appears promising, it is not yet comprehensive, and further investigation is still needed. Furthermore, saliva-based testing often requires relatively large sample volumes, which can pose challenges, particularly for elderly patients or those with difficulty producing saliva.^85^

### Optical Detection Methods

Optical detection is based on translating target binding events into an optical output. Many molecular mechanisms can be responsible for this output, including dye displacement, redox reactions, conformational changes, and fluorescence quenching or enhancement. Spectroscopic or optical techniques are utilized to monitor these molecular mechanisms and, thus, confirm target binding.^52^ Similarly to electrochemical methods, detection can either be label-free, where the detected signal is generated directly via target/transducer interactions, or label-based, where the optical signal is generated indirectly via a labeling compound.^86^ A wide array of optical sensor types and readout methods can be utilized for PoC detection of PSA. These range from simple LFAs, which can be analyzed using the naked eye, to more complex techniques, such as spectroscopic analysis with surface plasmon resonance (SPR).^87,88^ This versatility, coupled with high sensitivity/specificity, portability, low-cost, and rapid measurement times, makes optical detection methods very popular for emerging PoC tests for PSA determination (summary presented in Table 2).

**Table 2:** Summary of Optical Sensors for PSA Detection^a^

Sensor Materials/Capture Probe^b^	Readout Method^c^	Assay Time (min)	Sample Volume (μL)	Linear Range (ng/mL)	LoD (ng/mL)	Samples^d^	Ref
AuNPs-GO/immunoassay	Naked eye	120	-	1 × 10^–5^ to 0.1	3 × 10^–6^	Standard solutions	(89)
Ab					5 × 10^–6^ (serum)	Patient serum	
Au nanodisk array	FOLSPR	15	50	0.0001–1	0.0013	Standard solutions	(90)
Ab						Patient serum	
MQB/LFA	DCF Analyzer	60	50	0.01–100	0.009	Standard solutions	(91)
Ab						Spiked FBS	
						Patient serum	
Imprinted Au 2D nanoarray/QDs	FM	120	30	10–100	0.01	Standard solutions	(92)
Ab							
GNRbiochip/MFS	UV–vis–NIR	50	50	0.05–25	0.05	Standard solutions	(93)
Ab						Patient serum	
MMPs/EIA	CL	60	50	0.1–30	0.1	Standard solutions	(94)
Ab						Patient serum	
QD-embedded-SiO_2_NPs/LFA	Smartphone	25	60	0.1–100	0.14	Standard solutions	(95)
-Ab						Patient serum	
PGMNPs/LFA	Naked eye	15	50	1–128	0.17	Standard solutions	(96)
Ab	MAR					Spiked serum	
						Patient serum	
SiO_2_@Au–Ag NPs/LFA	CCD	20	30	0.1–10	0.3	Standard solutions	(97)
Ab						Patient serum	
AuNP/LFA	Photo scanner	20	30	0.3–30	0.3 (serum)	Standard solutions	(98)
Ab						Spiked serum	
Pdot-CANP/LFA	Naked eye-light source	10	10	2–10	0.32 (serum)	Standard solutions	(99)
Ab	DSLR					Spiked serum	
						Spiked whole blood	
QD NB/LFA	Fluorescent TSR	15	40	0.25–100	0.33 (serum)	Standard solutions	(100)
Ab						Spiked FBS	
						Patient serum	
STV-AuNP/LFA	PGM	40	100	1–100	1.26	Standard solutions	(101)
Ab							
AuNP/LFA	Cube Analyzer	20	20	0.5–150	2.5 (serum)	Patient serum	(43)
Ab						Patient whole blood	
AuNP-SpA/LFA	Naked eye	10	70	37–420	20 (urine)	Standard solutions	(102)
Ab	TSR					Patient urine	
Optical fiber	CL-photon detector	35	-	0.0008–100	0.0003	Standard solutions	(103)
Peptide						Spiked serum	
Au chip	SPR	30	-	0.1–1000	0.1	Standard solutions	(88)
Peptide-MB							
MB/LFA	Naked eye	10	-	0.1–100	10	Standard solutions	(87)
Peptide							
LFA	Naked eye	10	50	500–50,000	2000	Standard solutions	(104)
Lectin							
Imprinted Au chip	SPR	60	-	0.1–50	0.091	Standard solutions	(105)
MIP						Patient serum	

a Unless stated otherwise, the presented LoD values are for measurements performed in standard solution.

b Enzyme immunoassay (EIA); Green fluorescent coumarin derivative nanoparticles (CANP); Gold nanorod (GNR); Lateral flow assay (LFA); Magnetic beads (MBs); Microfluidic system (MFS); Micromagnetic particles (MMPs); Magnetic-quantum dot nanobeads (MQBs); Nanobead (NB); Nanowire (NW); Polymer dot (Pdot); Poly(acrylic acid)-modified gold magnetic nanoparticles (PGMNPs); Quantum dot (QD); Staphylococcal protein A (SpA); Streptavidin (STV).

c Charge-coupled device (CCD); Chemiluminescence (CL); Dual-color fluorescent (DCF); Digital single-lens reflex camera (DSLR); Fluorescent microscope (FM); Fiber-optic localized surface plasmon resonance (FOLSPR); Magnetic assay reader (MAR); Personal glucose meter (PGM); Surface plasmon resonance (SPR); Test strip reader (TSR); Ultraviolet, visible, near-infrared spectroscopy (UV–vis–NIR).

d Fetal bovine serum (FBS).

**Figure 2:**
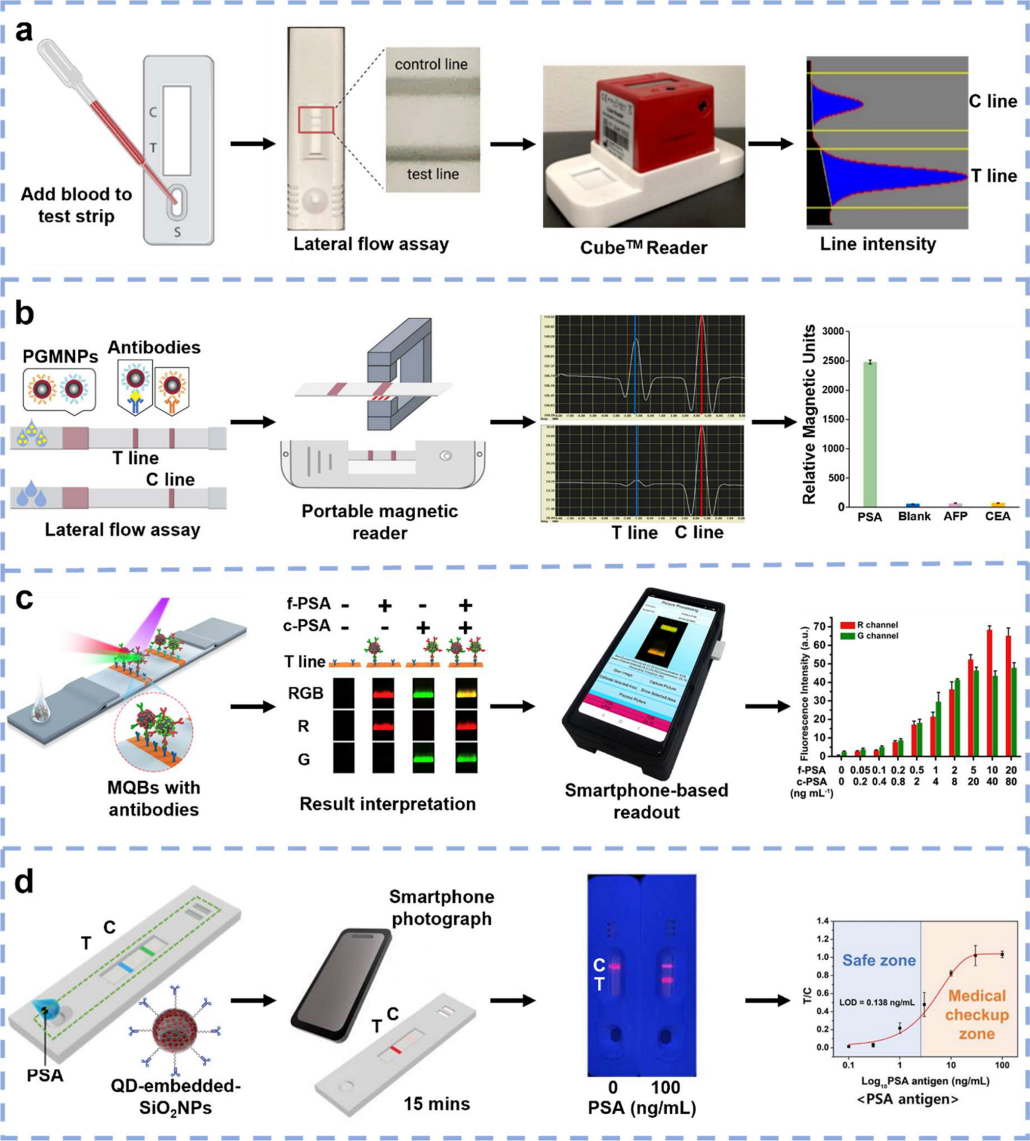
Emerging sensors for optical detection of PSA. (a) Whole blood is directly added to a LFA in which antibody-modified AuNPs are immobilized to the conjugate pad. The optical density of the test/control lines is analyzed using a portable Cube reader. Adapted from ref (43). Creative Commons CC BY license, Elsevier. (b) Magnetic AuNPs modified with antibodies are immobilized to the conjugate pad of an LFA and a magnetic assay reader is utilized for quantitative PSA determination. Adapted with permission from ref (96). Copyright 2021 Elsevier. (c) A fluorometric immunochromatographic test strip (ICTS) is developed for the simultaneous detection of fPSA and tPSA using dual-color magnetic-quantum dot nanobeads (MQBs) conjugated with antibodies. Quantitative determination is performed by using a smartphone-based dual-color fluorescent lateral flow strip reader. Adapted with permission from ref (91). Copyright 2019 Elsevier. (d) Antibody-modified SiO_2_NPs embedded with quantum dots (QDs) are immobilized to the conjugate pad of an ICTS and a smartphone-based reader is utilized for PSA determination. Adapted from ref (95). Creative Commons CC BY license, MDPI.

Pan et al. prepared Au biochips coated with antibodies, which could monitor PSA in the clinically relevant range (0.05–25 ng/mL) using a standard ELISA reader for signal analysis. The system showed a high correlation with a conventional ELISA assay, demonstrating a proof-of-concept for serum samples. However, the measurement time (∼50 min) and need for a microplate reader, which is not standardly available in laboratories, limits PoC application.^93^ LFAs are paper-based PoC diagnostic tools that have rapidly grown in popularity, as their low-cost, straightforward operation, and rapid measurement times overcome many drawbacks associated with ELISA-based systems. Traditionally, LFAs use antibodies as recognition elements combined with nanomaterials to enhance detection. Standard detection is in blood samples (whole blood or serum), but Di Nardo et al. explored PSA detection in urine with the naked eye.^102^ However, as there is no unanimous consensus regarding urine PSA tests in routine clinical practice, their application remains limited. Andreeva et al. developed an LFA with AuNPs to detect PSA levels in spiked serum samples. The intensity of the colored test lines was utilized to determine PSA levels in a range from 0.3–30 ng/mL with a photoscanner.^98^ Srinivasan et al. extended this approach to patient serum samples using AuNPs conjugated with antibodies to facilitate rapid PSA sensing (∼20 min). Quantification was enabled with a portable Cube reader, which measured the optical density of the test and control lines (Figure 2a). A high correlation (r = 0.95) for archived serum samples was found between the technology and the standard IMMULITE total PSA immunoassay. However, the cost of the portable Cube reader (∼$600) is too high for home testing.^43^

Attempts have been made to improve the sensitivity of LFAs for PSA by adapting the nanomaterial component to further enhance the measurement signal. For instance, magnetic AuNPs modified with antibodies were employed, which could detect PSA at 0.17 ng/mL but required a portable magnetic reader (Figure 2b).^96^ Furthermore, instead of pure Au, an Au–Ag alloy was assembled onto SiO_2_NPs to enable visual analysis of PSA. It was possible to semiquantitatively detect PSA concentrations in the range associated with early diagnosis to prognosis in clinical samples.^97^ Zhu et al. developed a bifunctional complex of enzyme (invertase) with antibodies conjugated onto AuNPs. Following the immunoreaction, the test and control lines were cut and placed onto a hydrophobic plate, where a sucrose solution was added to each zone. The invertase enzyme catalyzed the hydrolysis of sucrose to produce a specific amount of glucose, which was quantitatively determined with a commercial personal glucose meter. However, further engineering is required to achieve a one-step process and integrate the generation of invertase on testing lines with the readout of glucose production. Another significant drawback is that the nanozyme complex stability is limited to only 3 weeks.^101^

Currently, LFAs are most suited to qualitative diagnostic tests and do not have the capability for multimarker analysis. Fluorometric immunochromatographic test strips (ICTSs) can overcome these barriers by enabling target quantification using fluorophores. An example of multimarker analyte sensing was achieved by Rong et al., who employed dual-color magnetic quantum dots (QDs) conjugated with antibodies to measure fPSA and tPSA simultaneously. QDs are significantly brighter than other fluorescent NPs and have no photobleaching characteristics, which makes them inherently suitable for use in ICTS. A portable smartphone-based readout with low-cost optical and electrical components was developed to monitor the binding of the antibody-conjugated QDs in an ICTS format (Figure 2c). Despite favorable sensor performance, measurement times (60 min) were considerably longer than standard LFAs.^91^ A shorter assay time (15 min) was achieved by Li et al., who used highly stable QD-antibody conjugates to determine PSA concentrations in 40 μL samples. However, clinical potential is limited as the LoD in serum was 0.33 ng/mL, which is above the cutoff to determine BCR.^100^ Bock et al. utilized QDs embedded into SiO_2_NPs in an ICTS format to determine PSA levels via a smartphone and ImageJ analysis (Figure 2d). This device is highly convenient and showed no false-negative results in 47 patient serum samples. Nonetheless, there were some false-positive results in PSA concentrations of <2.5 ng/mL, which could have serious clinical implications.^95^

Bright semiconducting polymer NPs (Pdots) can be used as an alternative to QDs in ICTS. Yang et al. presented the first traffic light-like ICTS based on Pdots with multiplexing ability. The test line consisted of PSA (capture)-functionalized coumarin-based NPs and the control line consisted of bare coumarin-based NPs and antibodies. The porous membrane blocked blood cells; thus, the test could measure whole blood samples, as only plasma/serum could migrate via capillary flow. In the presence of PSA, fluorescence resonance energy transfer occurred between the coumarin-based NPs and Pdots, which led to an emission color transmission from sky blue to orange red. Therefore, results could be quickly (∼10 min) analyzed by using the naked eye under a portable 410 nm flashlight. This system was not used with patient samples or for quantitative determination. However, a more precise analysis of target concentration could be achieved by measuring the emission ratios of the test line to control line(s).^99^

Within optical sensors, there is also an ongoing search to replace antibodies with low-cost and robust alternative recognition elements. *Maackia amurensis* lectin II can recognize the terminal α-2,3 sialylation of PSA and was used in a proof-of-concept LFA. However, the LoD was 2000 ng/mL, which does not meet the required clinical specificity for PSA testing.^104^ Suaifan et al. prepared a PSA-specific peptide covalently bound to magnetic beads for highly selective binding. In the presence of proteolytically active PSA, the black magnetic carrier complexes were removed from the surface, which exposed the gold color sensor to the naked eye. The sensor could quantitatively detect PSA concentrations, but the LoD (10 ng/mL) was also insufficient for clinically relevant detection.^87^ Thus, antibodies are expected to remain the dominant recognition element for PSA detection with LFAs and ICTSs. Outside of the LFA format, other immunoassays require a significantly longer analysis time. For example, Liu et al. developed a magnetic-particle-based CLEIA that could detect PSA in the relevant diagnostic gray zone (LoD = 0.1 ng/mL). Microplate CLEIA typically requires 4–5 h, but this assay was considerably faster (∼1 h).^94^ In another study, a significantly lower LoD (5 × 10^–6^ ng/mL in serum) was achieved when a colorimetric immunoassay based on glucose oxidase-catalyzed growth of small AuNPs was employed instead of a CLEIA. The signal generated in the assay was similar to a standard horseradish peroxidase (HRP)-based ELISA where a colorless to yellow transition is observed, which improved quantitative detection. However, the assay time of 120 min is less suitable for detection at the time and place of the patient; thus, a LFA format is more promising for PoC tests.^89^

SPR is another emerging technology that, in contrast to LFAs, can be performed with a range of (biomimetic) recognition elements. The principle of SPR is based on measuring refractive index changes at the surface of a functionalized sensor chip when an analyte binds to a recognition element. Advantages of SPR biosensors include real-time and fast measurements, high sensitivity/specificity, and no need for labeled reagents.^106^ However, compared to LFAs, SPR is significantly more expensive, not available in all laboratories, and lacks portability. The straightforward functionalization of SPR chips has allowed researchers to utilize numerous recognition elements for PSA detection. For example, a self-assembled monolayer with magnetic beads conjugated with peptide was used to specifically detect a wide range (0.1–1000 ng/mL) of proteolytically active PSA.^88^ Ertürk et al. modified a SPR chip with surface imprinted polymers specific for PSA to evaluate clinical samples. Diluted patient serum samples (1:4 with buffer, n = 10) were measured, and results were benchmarked against a commercial ELISA kit with high correlation (linearity of 98%). Although the sensor was high-cost, it could be reused and a similar level of PSA detection was retained for 50 consecutive analyses.^105^ Kim et al. combined fiber optics with SPR to produce a miniaturized and low-cost device that facilitated remote sensing. Using Au nanodisk-antibodies, PSA was measured in buffered solutions and serum samples in a wide linear range (0.0001–1 ng/mL). While this enables detection at low concentrations, it does not cover the full clinical range required for monitoring of PCa.^90^ A wider linear range (0.0008–100 ng/mL) was obtained by Zhao et al., who developed a peptisensor that used biotinylated peptide-modified optical fibers and HRP-modified AuNPs to enable chemiluminescent detection. The test demonstrated high sensitivity in spiked serum samples and was low-cost with straightforward operation. However, no measurements were performed on clinical samples, assay time (∼35 min) was longer than LFAs, and considerable design changes are required to miniaturize the setup.^103^

### Commercial Point-of-Care Tests

In recent years, several PoC sensors for PSA determination have become commercially available (Table 3). All tests within Table 3 utilize optical detection (primarily LFAs), where the readout is performed by the naked eye or with an analyzer. The tests are portable, can measure whole blood, and have rapid assay times (10–20 min). Therefore, this allows measurements to be performed at the time and place of the patient with minimal sample preparation. Furthermore, sample volume requirements are low (35–80 μL), which facilitate minimally invasive finger prick methods for sample collection. However, despite these clear advantages, some of the tests have narrow measurement ranges and poor sensitivity and specificity, which may limit their widespread adoption by healthcare systems globally.

**Table 3:** Summary of Commercial PoC Tests for PSA Detection

Company/Device Name	Test Type	Assay Time (min)	Sample Volume (μL)	Range (ng/mL)	Sensitivity/Specificity (%)	Samples	Cost (USD)	Ref
CTK Biotech/*Onsite* Rapid Test	LFA	10	70	4–10	100/99	Whole blood	Cassette - 13	(107)
						Serum		
						Plasma		
AllTest/PSA Rapid Test	LFA	10	80	3–10	99.0/99.2	Whole blood	Cassette - 2	(108)
						Serum		
						Plasma		
PRIMA Lab/Prostate PSA Test	LFA	10	75	±4	97.2/87.1	Whole blood	Cassette - 12	(109)
Concile GmbH/CancerCheck PSA	LFA – Concile Ω100 Analyzer	20	75	0.5–25	85.7/66.7	Whole blood	Cassette -	(59, 110)
							Analyzer -1600	
OPKO Diagnostics/Sangia	Immuno-assay – Claros 1 Analyzer	12	35	0.08–15	85.4/30.3	Whole blood	Cassette - 15	(111, 112)
							Analyzer - 2500	
Boditech/i-CHROMA	LFA – Fluorescent Analyzer	15	75	0.1–100	–/–	Whole blood	Cassette - 7	(113)
						Serum	Analyzer - 2200	
						Plasma		

The *Onsite* PSA Semiquantitative Rapid Test (CTK Biotech) is a three-line LFA (control, test, and reference lines). The test is relatively low-cost (∼$13) and readout is performed with the naked eye in 10 min. Semiquantitative detection is achieved as no test line indicates PSA levels <4 ng/mL, a visible test line lighter than the reference indicates 4–10 ng/mL, and a test line darker than the reference indicates >10 ng/mL. Although the test has excellent sensitivity (100%) and specificity (99%), it is only within a narrow measurement range. Therefore, while it can act as a low-cost and rapid tool to screen for potentially elevated PSA levels, a laboratory-based immunoassay is still required.^107,114^ The PSA Rapid Test (AllTest Biotech) is a similar three-line LFA (reference at 10 ng/mL), which also utilizes the naked eye for a readout in 10 min. The test has a marginally larger measurement range (3–10 ng/mL) and very similar sensitivity (99.0%) and specificity (99.2%) compared to the CTK Biotech LFA. However, the test is low-cost ($2), which presents a key advantage for widespread use, particularly in resource-limited settings.^108^ The prostate PSA test (PRIMA Lab) is another LFA with a naked eye readout and 10 min assay time, but it has no reference line. Consequently, results only indicate PSA levels above or below 4 ng/mL. Furthermore, the sensitivity (97.2%) and specificity (87.1%) are also lower than the CTK and AllTest LFAs.^109^

Some commercial PoC tests utilize analyzers as a readout method to quantitatively detect PSA across wider ranges. However, this typically increases test cost/measurement time, reduces portability, and can lead to lower sensitivity/specificity compared to semiquantitative LFAs. The CancerCheck PSA test (Concile GmbH) is an LFA that uses a Concile Ω100 Analyzer (∼$1500) to quantitatively measure PSA levels in whole blood from 0.5 to 25 ng/mL in 20 min. Although the analyzer facilitates measurements across the clinically relevant range for screening, the sensitivity (85.7%) and specificity (66.7%) are somewhat limited.^59,110^ The Sangia Total PSA Test (OPKO Diagnostics) was the first FDA-approved (2019) PoC test for PSA determination. It is a microfluidic-based immunoassay (cassette = $15) where readout is performed using a Claros1 Analyzer (∼$2500) in 12 min. The test can quantitatively determine PSA levels ranging from 0.08 to 15 ng/mL, but the sensitivity (85.4%) and specificity (30.3%) are limited.^111,112,115^ The i-CHROMA (Boditech) utilizes LFAs ($7) and a fluorescent analyzer (∼$2200) for the quantitative determination of PSA across a wide range (0.1–100 ng/mL) in 15 min.^113^ The sensitivity/specificity are not provided, but Beltan et al. showed a good correlation (r^2^ = 0.9664) with another commercial PSA test (Elecsys PSA Test – Roche Diagnostics).^116^

Although PoC PSA tests have the potential to improve PCa diagnosis in resource-limited settings, it is unlikely that the tests presented in Table 3 could be adopted for widespread clinical use in these environments. This is because the tests either have good sensitivity/specificity but very narrow measurement ranges or wider measurement ranges but poor sensitivity/specificity. Therefore, these drawbacks would significantly limit the potential of the tests to effectively contribute to the PCa diagnostic pathway. Furthermore, although the tests are cheaper than laboratory-based immunoassays, the costs of individual cassettes (≤$15) and readers (≤$2500) are still relatively high, which could also limit their use.^111,112^ All the tests utilize antibodies as recognition elements, which have poor environmental stability (e.g., temperature, pH). Consequently, the tests have strict storage conditions and a limited shelf life, which creates significant issues, particularly as resource-limited environments often have climates that regularly exceed these storage temperatures.^107,111^

## Conclusions and Future Perspectives

There is a clear need for PoC PSA tests within the diagnostic pathway for PCa. Their widespread adoption across healthcare systems would significantly reduce costs and shorten the therapeutic turnaround time. Furthermore, they present an invaluable tool for PCa diagnosis in resource-limited settings where specialized equipment is not always available. Therefore, within this review, we have comprehensively assessed the translational potential of emerging laboratory-level and commercial PoC tests for PSA determination.

Laboratory-level PSA tests generally use electrochemical or optical detection methods. The most common electrochemical readout methods for direct and indirect PSA determinations are EIS and DPV, respectively. These electrochemical sensors are often integrated into portable devices, such as smartphones, to allow testing at the time and place of the patient. They typically exhibit favorable LoD values (median of 0.001 ng/mL), wide linear ranges, and low sample volumes (median of 50 μL). However, their measurement time is relatively high (median of 40 min), and many tests have costly sensor components. Optical detection methods mostly utilize LFAs, where readout is performed with the naked eye (low-cost, inferior detection) or with an analyzer (higher cost, superior detection). Generally, optical methods exhibit larger LoD values (median of 0.12 ng/mL) and narrower linear ranges compared with electrochemical methods. However, their sample volumes are the same (median of 50 μL), and the measurement time is significantly lower (median of 23 min). Commercial PoC PSA tests exclusively utilize optical detection, primarily with LFAs. Although these commercial tests are rapid with small sample volumes, they can have narrow measurement ranges and poor sensitivity/specificity. Furthermore, the use of benchtop analyzers for quantitative determination increases the cost and reduces portability. All commercial tests also use antibodies as recognition elements, which have some significant drawbacks, including low stability and high batch-to-batch variation. In contrast, ∼45% of laboratory-level tests use alternative recognition elements, such as aptamers, peptides, and MIPs, which have inherent advantages over antibodies.

Due to trade-offs between accuracy, accessibility, and affordability, no current PoC PSA tests (commercial or laboratory-level) meet all the REASSURED criteria. Although LFA-based optical PSA sensors are commercially available, their narrow measurement ranges or low sensitivity/specificity limit their potential to replace automated immunoassays. Consequently, we believe electrochemical sensors are the best suited to meet the REASSURED criteria for PSA tests. Specifically, we envisage an easy to operate smartphone-based electrochemical sensor that can provide accurate results across wide PSA ranges. Furthermore, the test will perform measurements in small volumes of whole blood, which will allow for minimally invasive, low-cost, and rapid finger prick sample extraction. Electrochemical microfluidic-based paper sensors appear promising for this application as they combine the advantages of LFAs (e.g., rapid, low-cost, ease of sample collection) with the high sensitivity/specificity of electrochemical detection. The test will be performed within a primary care setting in ≤20 min to allow testing and discussion of results within the same clinical appointment. Current laboratory-level electrochemical PSA sensors exhibit low LoD values and wide linear ranges, which make them suitable for clinical use. Moreover, potentiostats can link with smartphones to enable user-friendly operation with significantly lower equipment costs compared to automated immunoassays.

Despite having high sensitivity and user-friendly operation, current electrochemical sensors fail to meet the REASSURED criteria regarding rapid measurements, affordability (electrode cost), and ease of sample collection. To improve the measurement time, alternative recognition elements with high binding affinity can be explored, which can reduce incubation periods. Affordability can be optimized by using low-cost electrodes (e.g., SPCEs) and recognition elements (e.g., MIPs) within the sensors. To facilitate finger prick sample extraction (ease of collection) and testing in whole blood, emerging electrochemical tests should explore methods to incorporate blood separation membranes or microfluidics into their sensors. Another important consideration for test developers is creating standardized operating procedures and providing adequate reference materials. This will enable individuals to perform tests in a consistent, simple, and accurate manner with adequate quality control. Furthermore, it will allow comparisons of results from different test types and manufacturers in addition to benefiting the regulatory approval process. Despite the challenges related to developing PoC electrochemical PSA tests, research within this area is thriving, and sensors are constantly being improved. Therefore, we believe emerging electrochemical PSA tests will soon meet the REASSURED criteria and become an integral part of the PCa diagnostic pathway.

## Data Availability

No data was used for the research described in the article.

## Vocabulary

Prostate specific antigen: a protein produced by normal and malignant cells of the prostate gland that is commonly used as a key biomarker in the diagnostic pathway for prostate cancer
screening: a systematic process of testing asymptotic individuals to identify early signs of disease
point-of-care testing: performing tests and obtaining results at the time and place of a patient, generally outside of a laboratory setting
limit of detection (LoD): the minimum concentration of an analyte that can be reproducibly detected above the background noise or signal variability
electrochemical detection: a technique involving measuring electrical signals generated during chemical reactions at a sensor’s surface to detect analytes
optical detection: a technique involving measuring changes to light properties or color to detect analytes
